# Meta-analysis and suggested guidelines for prevention of venous thromboembolism (VTE) in foot and ankle surgery

**DOI:** 10.1007/s00167-015-3976-y

**Published:** 2016-03-18

**Authors:** James D. F. Calder, Richard Freeman, Erica Domeij-Arverud, C. Niek van Dijk, Paul W. Ackermann

**Affiliations:** The Fortius Clinic, London, UK; The Chelsea and Westminster Hospital NHS Trust, Imperial College, London, UK; Orthopaedic Department, Danderyd Hospital AB, Stockholm, Sweden; Orthopaedic Department, Amsterdam Medical Centre, Amsterdam, The Netherlands; Orthopaedic Department, Karolinska University Hospital, Stockholm, Sweden; Institution of Molecular Medicine and Surgery, Karolinska Institutet, Stockholm, Sweden

**Keywords:** Deep vein thrombosis, Foot and ankle surgery, Venous thromboembolism, Low molecular weight heparin, Achilles tendon, Lower limb surgery

## Abstract

**Purpose:**

To perform a meta-analysis investigating venous thromboembolism (VTE) following isolated foot and ankle surgery and propose guidelines for VTE prevention in this group of patients.

**Methods:**

Following a PRISMA compliant search, 372 papers were identified and meta-analysis performed on 22 papers using the Critical Appraisal Skills Programme and Centre for Evidence-Based Medicine level of evidence.

**Results:**

43,381 patients were clinically assessed for VTE and the incidence with and without chemoprophylaxis was 0.6 % (95 % CI 0.4–0.8 %) and 1 % (95 % CI 0.2–1.7 %), respectively. 1666 Patients were assessed radiologically and the incidence of VTE with and without chemoprophylaxis was 12.5 % (95 % CI 6.8–18.2 %) and 10.5 % (95 % CI 5.0–15.9 %), respectively. There was no significant difference in the rates of VTE with or without chemoprophylaxis whether assessed clinically or by radiological criteria. The risk of VTE in those patients with Achilles tendon rupture was greater with a clinical incidence of 7 % (95 % CI 5.5–8.5 %) and radiological incidence of 35.3 % (95 % CI 26.4–44.3 %).

**Conclusion:**

Isolated foot and ankle surgery has a lower incidence of clinically apparent VTE when compared to general lower limb procedures, and this rate is not significantly reduced using low molecular weight heparin. The incidence of VTE following Achilles tendon rupture is high whether treated surgically or conservatively. With the exception of those with Achilles tendon rupture, routine use of chemical VTE prophylaxis is not justified in those undergoing isolated foot and ankle surgery, but patient-specific risk factors for VTE should be used to assess patients individually.

**Level of evidence:**

II.

## Introduction

25,000 people die each year in England from venous thromboembolism (VTE), more than the combined total of deaths from breast cancer, AIDS and road traffic accidents [28]. The total cost (direct and indirect) to the UK for managing VTE is estimated at £640 million [28]. VTE has been highlighted as a particular risk following orthopaedic surgery or injury to the lower limb. However, most studies investigating VTE are conducted in patients undergoing major orthopaedic surgery at or above the knee [4, 5, 12, 13, 17, 42, 49, 68, 72]. The risk of VTE for patients with isolated foot and ankle conditions, even with plaster cast immobilization, and the possible benefits of mechanical and chemical prophylaxis are poorly studied.

The NICE committee commissioned with assessment of VTE prevention concluded that for patients immobilized in a cast “This is a large patient group for whom the evidence is not clear” and went on to state “There would be a substantial cost to the NHS of providing thromboprophylaxis to all patients with a lower limb plaster cast, particularly if patients use prophylaxis until cast removal which may be a number of weeks” [53]. The American College of Chest Physicians (ACCP) most recent review also recommends against chemical prophylaxis in lower leg injuries requiring immobilization [15]. Despite this conclusion, many hospitals are introducing policies which recommend the routine use of low molecular weight heparin (LMWH) chemoprophylaxis for those in a cast following ankle fractures and all forms of elective foot and ankle surgery.

In order to make such recommendations, the following criteria must be fulfilled:

1. There is a significant risk of VTE in those with isolated foot and ankle conditions.
2. The incidence of VTE is significantly reduced by prescribing LMWH prophylaxis.
3. The risk of complications from LMWH outweighs the reduction in risk of VTE.
4. There is an appropriate cost–benefit using LMWH for VTE prophylaxis.

The purpose of this meta-analysis and review of the literature is to establish the incidence of VTE in orthopaedic foot and ankle patients, specifically investigating the effectiveness and risk of chemoprophylaxis comparing clinical to radiographic outcome measures.

The aim of the paper is to identify those factors that increase the risk of VTE in patients with foot and ankle conditions and establish whether current guidelines should be revised to consider preventive methods in all or specific patients undergoing foot and ankle surgery.

## Materials and methods

### Search strategy

A PRISMA compliant search of AMED, EMBASE, HMIC, MEDLINE, BNI and CINAHL databases on the 31 January 2015 was undertaken [51]. The search terms were: thromboembolism and (foot OR ankle) = 308 then combined with a search for: VTE and (foot OR ankle) = 64. Review of meeting abstracts and relevant references identified an additional 32 studies for potential inclusion. Fifty-two duplicates were removed and 328 articles excluded by title and abstract screening.

**Fig. 1:**
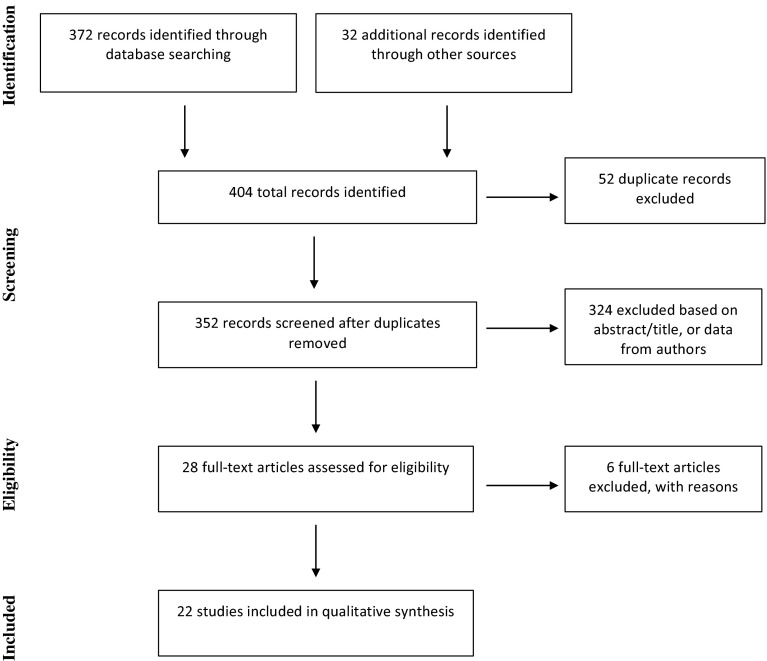
Preferred reporting items for systematic reviews and meta-analyses (PRISMA) flow diagram of article selection

The methodological quality of each article was assessed using the Critical Appraisal Skills Programme (CASP) [59]. The CASP checklist assessed whether the aim of the paper was clear, the methods were valid (including study design, recruitment, bias and ethics) and there were a rigorous analysis of data and a clear statement of findings. In total 28 articles were independently reviewed by two of the authors (RF and EDA) using the CASP tool and Centre for Evidence-Based Medicine (CEBM) level of evidence [55]. Any discrepancies were resolved by consensus with the senior author (JC). Twenty-two studies met full inclusion criteria for the final analysis (Fig. 1).

### Exclusions

- Case reports
- Non-original data, meta-analyses, etc.
- Evidence level 4 and below
- CASP score of 8 or less
- Patients with pathology proximal to the mid-tibia
- Studies with <12 patients per subgroup

### Assumptions and simplifications

For the purposes of the meta-analysis, all studies were considered as cohorts, such that an RCT with two arms was considered as two separate cohorts. Patient cohorts have been simplified into two categories: general foot and ankle patients including elective and trauma patients that may or may not have required a below knee cast and patients with acute Achilles tendon rupture treated with or without surgery. Achilles patients were considered in a separate meta-analysis as they have been found to have significantly higher risks of VTE in some studies, and these studies were clearly outliers in our provisional review of the data.

LMWH regimens were considered to be equal although formulations and length of treatment may vary. Two further studies, one using extended intermittent pneumatic compression devices (IPCDs) and one using aspirin, were also included in the prophylaxis group.

The principal study measures are VTE rates, a sum of the DVT and PE incidence.

Statistical advice was sought from the Biostatistics Unit, University College London, UK. Analysis was performed using STATA. The results are presented as incidence with 95 % confidence intervals. A meta-analysis was performed initially using a fixed effects model with a test for homogeneity. If homogeneity was unlikely (a pre-hoc probability of *p* = 0.2), then a random effects model was used.

## Results

### Narrative results

In total 22 studies met the criteria for inclusion: 10 of these assessed VTE clinically, 7 with ultrasound and 5 with venography.

### Patient populations

There was heterogeneity in the study populations with some studies offering data on various subgroups. Six studies considered general foot and ankle cases [16, 22, 24, 32, 36, 40]. Twelve studies had trauma cohorts [21, 33, 36–38, 41, 56, 57, 62, 66, 67, 69]. Six studies looked at Achilles injury, some of these included patients regardless of treatment whilst others focused specifically on Achilles surgery [9, 26, 30, 36, 54, 64].

### Prophylaxis

There was also some heterogeneity in prophylaxis regimens. Most studies used LMWH either in comparison to no prophylaxis [21, 32, 33, 36–38, 42] or in isolation [24, 57, 76]. One study compared aspirin with no prophylaxis [22] and another compared an Intermittent Pneumatic Compression Device with no prophylaxis [9]. The remaining studies used no formal post-operative prophylaxis [16, 26, 41, 54, 62, 64, 66, 67] or were unclear [30, 69].

### Methodological quality

The studies showed moderate-to-good methodologies according to the CASP appraisal tool. All studies were focused with an appropriate method and acceptable recruitment. The exposure was generally measured to minimize bias (21/22) although there was a risk of bias in the outcome measures of some (4/22). Confounding factors were identified in 14 of 22 studies and accounted for in the analysis of 15 of 23. Follow-up was considered complete enough in all but three of the studies (19/22), seven studies used large hospital databases to follow patients and 12 had over 80 % follow-up. The length of follow-up was 35 days or more in 11 studies, in a further seven studies using large hospital databases, it was assumed to be sufficient and in four was considered to be insufficient. All study populations were relevant, and the results were comparable to other studies in most cases (18/22). Overall, the authors were in favour of prophylaxis in seven studies, against in nine, and no clear conclusion was drawn in seven.

### Risk factors for VTE

Of the 12 studies that analysed risk factors for VTE, age was found to be a factor in six studies [32, 36, 37, 62, 66, 67]; no statistical association with any risk factor was shown in three studies [21, 56, 57]; injury severity was associated with risk of VTE in two studies [62, 66]; obesity was a factor in three studies [16, 37, 66] and immobilization was a factor in three studies [36, 62, 67]. Other risk factors found in individual studies were as following: non-weight bearing [62], hindfoot surgery [67], tourniquet time [67], varicose vein [37] Charlson score >2 [32], NIDDM [32], air travel [24], prior VTE [16], hormone replacement therapy (HRT) and oral contraceptives [16].

### Meta-analysis results

To reduce the risk of heterogeneity in the study design affecting the results, the following meta-analyses were performed:

- All studies using clinical indicators as the primary assessment of VTE—this group was divided into patients who either received or did not receive prophylaxis.
- All studies using radiological means as the primary assessment of VTE—again this group was divided into patients who either received or did not receive prophylaxis.
- Studies investigating VTE purely following Achilles tendon rupture were assessed separately as the high rates of DVT were obvious outliers when compared to other foot and ankle injuries or treatments [9, 26, 30, 40, 54, 64].
- Studies where the prophylaxis regimen was unclear were excluded [30, 69].

### Clinical assessment of VTE in patients with foot and ankle conditions

**Fig. 2:**
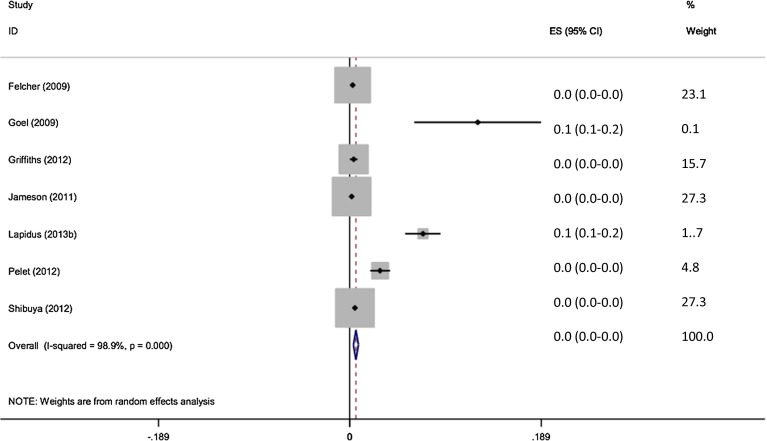
Forest plot of clinical assessment of VTE without prophylaxis

A total of 43,381 patients were clinically assessed for the presence of VTE. 126 of 27,139 patients without any form of prophylaxis developed VTE (0.46 %). The pooled effect size shows the incidence of VTE without prophylaxis to be 0.6 % (95 % CI 0.4–0.8 %) (Fig. 2).

**Fig. 3:**
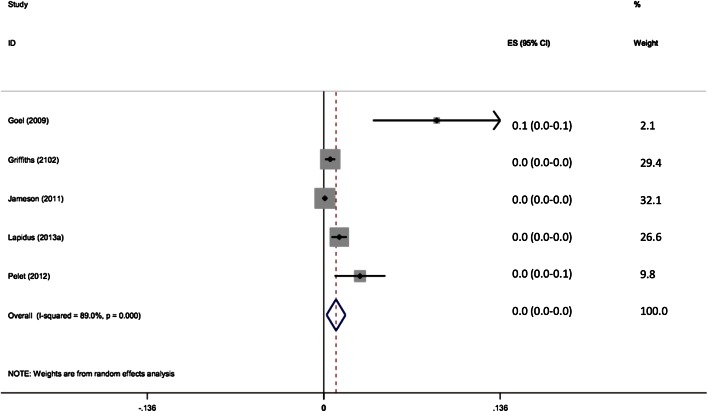
Forest plot of clinical assessment of VTE with prophylaxis

45 of 16,242 patients with prophylaxis developed VTE (0.28 %). The pooled effect size shows the incidence of VTE with prophylaxis to be 1 % (95 % CI 0.2–1.7 %) (Fig. 3). There was no significant difference in the rate of VTE between the groups with and without chemoprophylaxis.

### Radiological assessment of VTE in patients with foot and ankle conditions

**Fig. 4:**
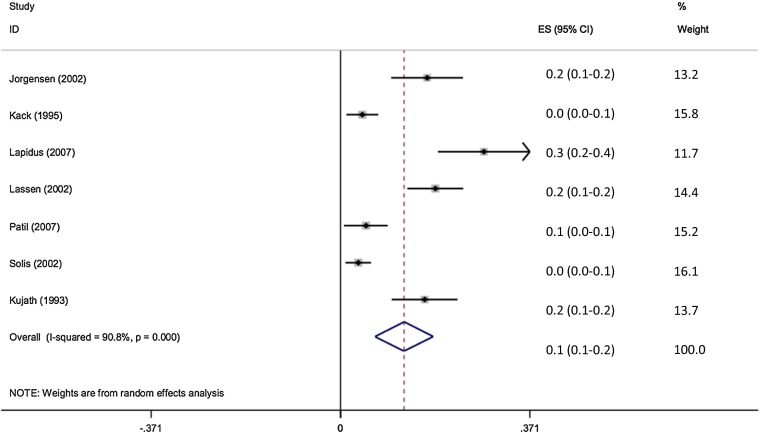
Forest plot of radiological assessment of VTE without prophylaxis

1666 Patients were assessed for radiological evidence of DVT. 120 of 981 patients without any form of prophylaxis developed VTE (12.2 %). The pooled effect size shows the incidence of VTE without prophylaxis to be 12.5 % (95 % CI 6.8–18.2 %) (Fig. 4).

**Fig. 5:**
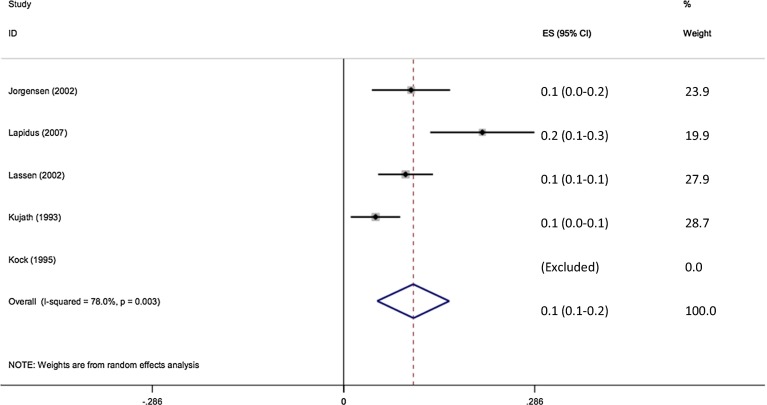
Forest plot of radiological assessment of VTE with prophylaxis

54 of 685 patients with prophylaxis developed VTE (7.9 %). The pooled effect size shows the incidence of VTE with prophylaxis to be 10.5 % (95 % CI 5.0–15.9 %) (Fig. 5). There was no significant difference in the rate of VTE between the groups with and without chemoprophylaxis.

### Patients with Achilles tendon rupture

**Fig. 6:**
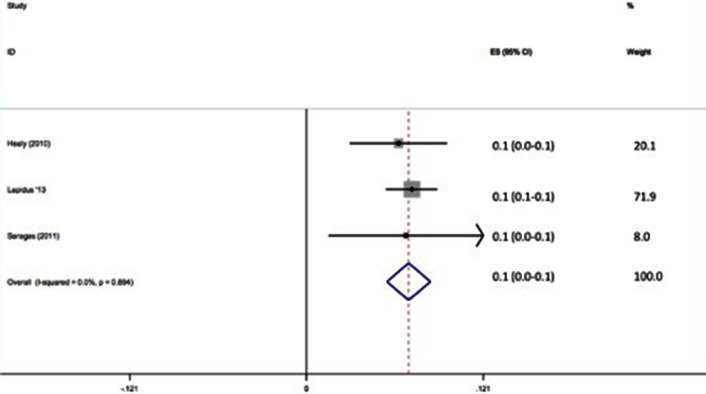
Forest plot of incidence of clinically assessed DVT in Achilles tendon rupture

1060 patients were assessed clinically for evidence of DVT, and 74 were confirmed to have VTE (7 %). The pooled effect size shows the incidence of VTE to be 7 % (95 % CI 5.5–8.5 %) (Fig. 6).

**Fig. 7:**
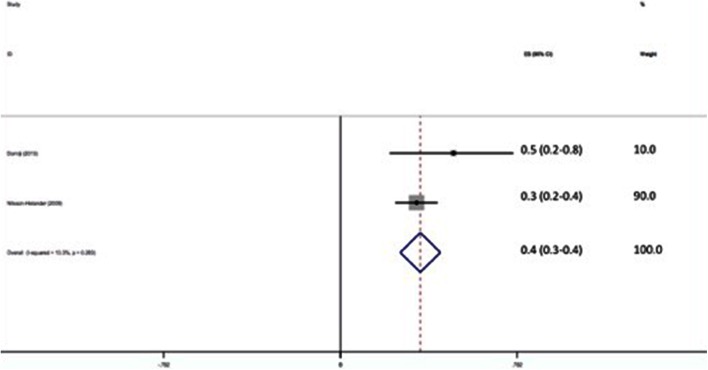
Forest plot of incidence of radiologically assessed DVT in Achilles tendon rupture

Hundred and seven patients were assessed for radiological evidence of DVT and 38 were confirmed to have VTE (35.5 %). The pooled effect size shows the incidence of VTE to be 35.3 % (95 % CI 26.4–44.3 %) (Fig. 7).

Only one RCT reported the effect of LMWH on rate of VTE following immobilization of 105 patients with Achilles tendon rupture. There was no significant reduction in the rate of DVT with 34 % in the LMWH group and 36 % in the control group [42].

## Discussion

The most important finding of this study is that there is a low risk of developing VTE following isolated foot and ankle surgery and no benefit could be demonstrated by using chemoprophylaxis. The incidence of VTE without prophylaxis was 0.6 % when diagnosed clinically and 12.2 % with radiological diagnosis which is similar to the meta-analyses by Ettema et al. and Testroote et al. who both also reported on VTE following lower limb immobilization [14, 70, 71]. It is also similar to the background risk of spontaneous VTE of 0.2–0.5 % [18, 25]. This is lower than in general orthopaedic surgery where the rate of DVT is reported as 40–60 % [23], but similar to the incidence of DVT following knee arthroscopy which has been reported as 0.6 % when diagnosed clinically and up to 17.9 % when using radiography [31, 60]. The consequences of asymptomatic below knee DVT and the importance of its prevention and treatment remains controversial and a systematic review of the treatment of below knee DVT’s concluded there was insufficient evidence to recommend treatment over mere surveillance [8, 19, 29, 34, 43, 45, 58, 61, 74].

Various methods of prophylaxis may be employed but no method completely protects against VTE [35]. LMWH is the current standard by which other chemical agents are compared. However, the ideal duration of treatment has yet to be confirmed in orthopaedic surgery with some protocols advocating treatment only whilst in hospital and others whilst immobilized or for an arbitrary period ranging from 2 weeks to 35 days.

Out of hospital, compliance rates may drop below 85 % and oral agents may increase patient compliance [15, 47, 73]. The ACCP review concluded that a 160 mg dose of aspirin for 35 days following lower limb injuries would prevent 7 per 1000 VTE’s but at the expense of three major bleeding episodes and two non-fatal myocardial infarctions [15]. Only one paper investigated the use of aspirin in foot and ankle surgery, and no benefit in protecting patients from VTE could be demonstrated [22]. To our knowledge, warfarin has not been investigated with regards to VTE prophylaxis in foot and ankle surgery. New oral anticoagulants such as dabigatran and rivaroxaban are only currently licensed for use following elective hip and knee arthroplasty, and to date no studies have investigated their use in patients undergoing foot and ankle surgery [48].

Therefore, if chemoprophylaxis is to be recommended it would appear that only LMWH has a body of evidence to support its use. However, this meta-analysis has failed to demonstrate any significant reduction in the risk of VTE with the use of LMWH in foot and ankle conditions irrespective of the method of assessment—clinical assessment 0.6 versus 1 % with prophylaxis (*p* = n.s.) and radiological assessment 12.5 versus 10.5 % with prophylaxis (*p* = n.s.).

In addition to the lack of effectiveness of chemoprophylaxis following isolated foot and ankle surgery, it is recognized there are potential risks of administering LWMH. These risks include bleeding (0.3–1 % following lower limb surgery) [17, 42, 46, 52], bruising and haematomas (12 %) [33, 42], wound healing problems and increased rate of wound infection, of particular concern in the foot and ankle [33, 36, 42]. Heparin-induced thrombocytopenia (HIT) is a potentially life-threatening adverse effect, more common in post-operative patients than medical patients with a rate of 2.6–6.5 % using unfractionated heparin and 0.2–0.35 % with LMWH [6, 20, 44].

The risk of developing VTE in the subgroup of patients with Achilles tendon rupture appears to be particularly high whether treated surgically or non-operatively. Nilsson-Helander et al. and Lapidus et al. both reported an incidence of 36 % when screened with USS [39, 54]. We separated Achilles tendon rupture patients from the main analysis as the results were clear outliers when compared with other foot and ankle conditions with few good-quality studies and only a small number of patients. It may be that because of direct involvement and de-functioning of the gastro-soleus complex, Achilles tendon ruptures need to be considered separately from general foot and ankle cases with regards to VTE prophylaxis. Although LMWH has been shown to have little or no effect in prevention of DVT following Achilles rupture [39], a recent RCT of 150 patients using mechanical IPCDs for 2 weeks following Achilles tendon repair has demonstrated an absolute risk reduction for DVT of 37–21 % in the treated group (OR 2.60; 95 % CI 1.15–5.91; *p* = 0.022) [10]. Active mechanical methods address the problems of stasis, and further research into this area is justified.

This meta-analysis could be criticized for including studies with a wide variety of foot and ankle cases including both elective and trauma. This trade-off increases the numbers included in the analysis at the expense of some clinical heterogeneity. However, we believe it represents the realities of clinical practice. The studies were also statistically heterogeneous which reflects differences in study protocols, and we recognize that this heterogeneity limits the interpretation of any study on VTE rates in this population.

With the exception of age, the studies included in this review show conflicting results regarding risk factors such as restricted weight bearing, obesity and smoking where some studies report an association with VTE [3, 11, 50]. However, there was too much study heterogeneity to specifically investigate the effect of individual risk factors. A history of previous VTE and thrombophilia has been shown to significantly increase the risk of further VTE with a 23 % 5-year rate of recurrence of proximal DVT, 6 % for calf DVT and pulmonary embolus 3–4 times as likely to recur in a meta-analysis by Baglin et al. [1, 2].

Previous studies of chemical prophylaxis and RCTs of LMWH for lower limb immobilization have reported insignificant effects in the prevention of DVT [14, 22, 38, 65]. A retrospective study of 664 total ankle replacements reported a clinical VTE rate of 0.6 % without prophylaxis, unless there was a previous history of VTE and a recent double-blind RCT of the effects of LMWH following surgery and immobilization for lower leg fractures was stopped after interim analysis of 258 patients demonstrated an incidence of clinical VTE of 1.9 % and no significant benefit of using chemical prophylaxis [27, 65].

This paper would support the view that the risk–benefit of chemoprophylaxis for those with isolated foot and ankle conditions should be assessed separately from those undergoing general lower limb orthopaedic surgery. Although there was inconsistency in their effect on VTE risk in foot and ankle surgery, undoubtedly certain patient-related factors increase the risk of VTE including smoking, obesity, age >60 years, malignancy, HRT, oral contraception, previous VTE and thrombophilia and these should continue to be taken into account when assessment is made as to the need for chemoprophylaxis. It is also recognized that multiple risk factors are cumulative and two or more risk factors may lower the threshold for considering the benefit of chemoprophylaxis over the risks and costs of its use [7, 63]. Mechanical methods such as TEDS and IPCDs may be a targeted alternative to chemoprophylaxis for DVT prevention in lower limb-immobilised patients after foot and ankle surgery. These patients should also routinely be encouraged to mobilize early and avoid dehydration (Table 1).

**Table 1:** Characteristics of included studies

Author (year) [reference]	Study design	Number of patients	Patients	Detection method	CASP score (mean)
Domeij (2013) [10]	RCT	24	DVT 2 and 6 weeks following surgery for Achilles rupture—IPCD versus no prophylaxis	DVT—US	9
Felcher (2009) [16]	Retrospective cohort	7264	Database search for VTE within 6 months of surgery	DVT—USS PE—VQ/CTPA scan	10
Goel (2009) [21]	RCT	238	LMWH versus placebo following surgery for below knee fractures	DVT—venography	10
Griffiths (2012) [22]	Case control	2654	75 mg aspirin versus no chemical prophylaxis	Symptomatic VTE	9.5
Hanslow (2006) [24]	Retrospective cohort	608	Foot and ankle surgery (high-risk patients received LMWH)	Symptomatic VTE	8.5
Healy (2010) [26]	Retrospective cohort	208	Achilles rupture (cast and surgery) no chemoprophylaxis	Symptomatic VTE confirmed by USS/CTPA	10
Ingvar (2005) [30]	Retrospective cohort	196	Achilles rupture treated conservatively	Symptomatic VTE	8
Jameson (2014) [32]	Retrospective cohort	88,241	Database search for VTE before and after introduction of NICE guidelines	Hospital episode statistics	10.5
Jorgensen (2002) [33]	RCT	300	Below knee cast immobilization—LMWH versus no prophylaxis	DVT—venography	9
Kock (1995) [36]	RCT	339	Below knee cast immobilization—LMWH versus no prophylaxis	DVT—USS confirmed with venography	10.5
Kujath (1993) [37]	RCT	253	Below knee cast immobilization—LMWH versus no prophylaxis	DVT—USS PE—VQ	12
Lapidus (2013) [40]	Prospective cohort	5894	No routine prophylaxis for foot and ankle surgery except LMWH for ankle fractures	DVT—USS PE—VQ/CTPA scan	11
Lapidus (2007) [38]	RCT	272	Ankle fractures—LMWH until cast removal versus no prophylaxis	DVT—venography	10.5
Lassen (2002) [41]	RCT	440	Ankle fractures—LMWH until cast removal versus no prophylaxis	DVT—USS PE—VQ/CTPA scan	10.5
Nilsson-Helander (2009) [54]	RCT	95	Surgery versus no surgery for Achilles rupture—no routine prophylaxis	DVT—USS PE—VQ/CTPA scan	9
Patil (2007) [56]	Prospective cohort	100	Below knee cast immobilization for ankle fractures—no routine prophylaxis	DVT—USS	10.5
Pelet (2012) [57]	Retrospective cohort	1540	Surgery for ankle fracture—no routine prophylaxis (141 low dose aspirin; 253 LMWH)	Symptomatic VTE confirmed by USS/VQ/CTPA scan	11
Riou (2007) [62]	Prospective cohort	2757	Below knee cast immobilization—chemoprophylaxis versus no prophylaxis	DVT—USS	10.5
Shibuya (2012) [66]	Retrospective cohort	75,664	Database search for foot and ankle trauma	Symptomatic VTE	10.5
Saragas (2011) [64]	Retrospective cohort	88	Surgical repair Achilles rupture—no prophylaxis	Symptomatic VTE confirmed by USS	8.5
Solis (2002) [67]	Prospective cohort	201	No routine prophylaxis for foot and ankle surgery	DVT—USS	9
Soohoo (2011) [69]	Retrospective cohort	57,183	Database search for ankle fractures undergoing surgery	Readmission for VTE	9

*RCT* randomized controlled trial, *IPCD* intermittent pneumatic compression device, *VQ/CTPA scan* ventilation-perfusion/computerized tomographic pulmonary angiography scan

The findings of this meta-analysis are summarized using a Grade of Recommendation (Tables 2, 3), and guidelines for considering VTE prophylaxis in isolated foot and ankle conditions is proposed in Table 4 [75].

**Table 2:** Grades of recommendation for orthopaedic surgical studies

Grade of recommendation	Description
A	Good evidence (Level I studies with consistent findings) for or against recommending intervention
B	Fair evidence (Level II or III studies with consistent findings) for or against recommending intervention
C	Poor quality evidence (Level IV or V studies with consistent findings) for or against recommending intervention
I	There is insufficient or conflicting evidence not allowing a recommendation for or against intervention

**Table 3:** Grade of recommendation assigned summarizing main findings of the meta-analysis

Routine chemoprophylaxis is not indicated for patients undergoing isolated foot and ankle surgery (Grade A recommendation)
Routine chemoprophylaxis is not indicated for patients with restricted weight bearing or immobilized for isolated foot and ankle conditions (Grade B recommendation)
Routine use of mechanical anti-VTE methods is indicated following Achilles tendon rupture whether treated surgically or non-operatively as there is a higher risk of VTE (Grade B recommendation)
Chemoprophylaxis with LMWH should be considered if two or more risk factors (smoking, obesity, age >60 years, malignancy, HRT, oral contraception, previous VTE and thrombophilia) are present in patients with isolated foot and ankle conditions (Grade C recommendation)

**Table 4:** Suggested guidelines for prevention of VTE in routine isolated foot and ankle surgery (with/without immobilization and reduced weight bearing)

Start mechanical VTE prophylaxis at admission using one of the following:
Anti-embolic stockings (thigh or knee length)—assuming no contraindications
Foot impulse devices
Intermittent pneumatic compression devices (thigh or knee length)
If patient has a history or previous VTE/thrombophilia or two or more risk factors below consider chemical prophylaxis (LMWH commencing 6–12 h after surgery until discharge from hospital or if immobilized and/or reduced weight bearing continue until the patient no longer has significantly reduced mobility)
Active cancer or cancer treatment
Age over 60 years
Smoking
Critical care admission
Dehydration
Obesity [body mass index (BMI) over 30 kg/m^2^]
Use of hormone replacement therapy
Use of oestrogen-containing contraceptive therapy
Varicose veins with phlebitis

## Conclusion

The incidence of clinically apparent VTE following foot and ankle surgery is less than 1 % without using chemical prophylaxis, and no benefit could be demonstrated by using LMWH. Routine chemoprophylaxis cannot be recommended following isolated foot and ankle surgery. The one group where there may be a significant risk of VTE is following Achilles tendon rupture when specific preventative measures such as IPCDs may be indicated, and further research should investigate mechanical methods.
